# Development of peripheral eosinophilia in inflammatory bowel disease patients on infliximab treated at a tertiary pediatric inflammatory bowel disease center is associated with clinically active disease but does not result in loss of efficacy or adverse outcomes

**DOI:** 10.1002/jgh3.12308

**Published:** 2020-02-07

**Authors:** Douglas Zabrowski, Danielle Abraham, Geoffrey Rosenthal, Howard Kader

**Affiliations:** ^1^ Department of Pediatrics, Division of Pediatric Gastroenterology/Nutrition University of Maryland School of Medicine Baltimore Maryland USA; ^2^ Department of Epidemiology and Public Health University of Maryland School of Medicine Baltimore Maryland USA; ^3^ Department of Pediatrics, Division of Cardiology University of Maryland School of Medicine Baltimore Maryland USA

**Keywords:** adverse events, complications, eosinophilia, inflammatory bowel disease, infliximab

## Abstract

**Introduction:**

Inflammatory bowel disease (IBD) consisting of Crohn's disease (CD) and ulcerative colitis (UC) are inflammatory conditions affecting the gastrointestinal tract. Infliximab (IFX) is a chimeric anti‐tumor necrosis factor antibody used to treat moderate to severe IBD. Eosinophils are commonly found in chronically inflamed tissues in IBD. Peripheral eosinophilia (PE) was previously implicated as a marker of disease severity at diagnosis. The main aim of this study was to investigate whether in IBD patients on IFX, development of PE is associated with adverse outcomes and poor IFX efficacy.

**Methods:**

A comprehensive retrospective chart review of IBD patients on IFX (January 2006 to July 2015) treated at a tertiary pediatric IBD center was performed. Data was collected at time specified points over a 24 month period and included demographics, atopy, disease severity, development of PE, human antichimeric antibodies (HACA), infusion reactions, cancer, psoriasis, and loss of clinical response.

**Results:**

One hundred twenty‐one IBD patients starting IFX (67 male), mean age of 12.4 years (range 4–22 years old), met inclusion criteria. Of them, 36.3% had ≥1 PE episode (CD: 25 male, 11 female; UC: 6 male, 2 female). Mean absolute eosinophil count (AEC) did not change over time. PE was associated with clinically active disease. Among patients who developed PE, adverse outcomes were not significantly different *versus* those who did not have PE.

**Conclusions:**

In a cohort of primarily pediatric IBD patients on IFX, PE was associated with clinically active disease; however, PE was not related to increased incidence of adverse outcomes or loss of drug efficacy.

## Graphical abstract

In pediatric Inflammatory bowel disease (IBD) patients on infliximab, peripheral eosinophilia was associated with clinically active disease, but was not related to increased incidence of adverse outcomes or loss of drug efficacy.

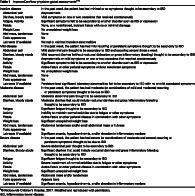

## Background

Crohn's disease (CD) and ulcerative colitis (UC) are the two major subtypes of inflammatory bowel disease (IBD), characterized primarily by chronic inflammation of the gastrointestinal tract that has a fluctuating course and that may be associated with systemic inflammation in other organ systems. The etiology of IBD is not completely understood, although immune dysregulation and genetic factors are believed to be significantly implicated.^1^, ^2^

Eosinophils, a subset of leukocytes, are sparsely present in healthy tissues and exist as a low percentage of total blood leukocytes. Excess eosinophils are usually associated with allergic/atopic conditions or parasitic infections.^3^ Eosinophils have been implicated in the pathogenesis of IBD by some investigators as they are found in excess in the cellular milieu of inflamed tissues of IBD patients distinct from other eosinophilic gastroenteritis.^4^, ^5^, ^6^ Products of eosinophil degranulation have been reported in excess in stools of patients with active IBD.^7^ Eosinophils have also been demonstrated in tissues as an adverse consequence of medications.^8^, ^9^, ^10^, ^11^ Previously, peripheral eosinophilia (PE) has been noted by Sadi *et al*. to be a possible indicator of increased disease severity at diagnosis of IBD.^12^

Infliximab (IFX) is a human‐mouse chimeric anti‐tumor necrosis factor antibody used in the treatment of moderate to severe IBD. Cutaneous eosinophilia has previously been reported as a rare adverse effect of IFX.^13^, ^14^ However, although eosinophils are implicated in IBD pathogenesis and eosinophilia may result from immune reaction to foreign proteins, the association between PE alone and adverse events and diminished efficacy of IFX has not been evaluated. Consequently, this retrospective study was conducted to determine whether episodes of PE, among patients on IFX, are related to adverse outcomes, primarily the development of human anti‐chimeric antibodies (HACA) and/or loss of clinical response to IFX.

## Methods

### *Study sample*

Following approval by the University of Maryland, Baltimore Institutional Review Board, a comprehensive medical chart review was performed for all patients with IBD treated by the University of Maryland's Children's Hospital Center's Pediatric IBD center who also received IFX from January 2006 through July 2015. Three patients aged between 18 and 22 years were included as they were also treated at this pediatric IBD center. Patient charts to be reviewed were identified from the Pediatric Infusion Center records. Patients with other known hypereosinophilic disorders, insufficient or incomplete medical records as defined by an incomplete IFX induction course, and a preponderance of missing lab values or clinical documentation, and those who were started on IFX prior to being treated at our center were excluded. This research study was approved of by the Institutional Review Board of the University of Maryland and was found to be exempt from full review.

### *Data collection and variables*

Variables collected at baseline included the subject's demographics, IBD subtype, IBD phenotype, disease location, atopic conditions (asthma, eczema, psoriasis, seasonal allergies, or food allergies), IBD‐related surgical history, and age at IFX initiation. Longitudinally collected data included laboratory data, physician global assessments, other IBD medications (antibiotics, corticosteroids, mesalamine, 6‐mercaptopurine, azathioprine, and methotrexate), and clinical outcomes (IFX dose increases, frequency of IBD flares, IFX infusion reactions, number of hospitalizations, and other IBD‐related morbidities).

Each chart was analyzed to obtain data from specified time points. Specified study time points included prior to the start of IFX (baseline), after first infusion (2 weeks), after completion of induction (10 weeks), and at 6 months, 1 year, and 2 years after starting IFX. Data were also collected at additional time points associated with adverse outcome visits around disease flares, infusion reactions, dosage increases, and HACA positive adverse events. For IFX dose increases, data were collected immediately prior to and after the first increased dose and again 6 months after the increased dose. All assessed time points were within 1 month of targeted time points. Full data from visits outside of these specified study points but still within the study period were reviewed for absolute eosinophil count (AEC), but not comprehensively analyzed. Data including all visit types listed will be referred to as “all visits”. If there were insufficient data at a specified visit, that visit was omitted from the analysis.

### *Eosinophilia*

Subjects with an AEC value greater than or equal to 500 cells/μL for at least one of the specified time points were classified as having had a PE event at that visit. Consecutive visits with PE without returning to a normal AEC value were defined as having PE. Patients with only one isolated PE lab value were also defined as having PE. Additionally, the frequency of unique, separate PE events at any visit over the study period, including those not related to adverse events or predetermined time points, were recorded.

### *Outcomes related to IFX*

Recorded and analyzed outcomes included a requirement for dose increase, the frequency of disease exacerbation, infusion reactions, number of hospitalizations, physician global assessment, and disease related complications. IBD exacerbation was defined as an acute change in symptoms requiring hospitalization, initiation of steroids, or a dose increase of IFX.

Physician global assessment (PGA) of disease severity was based on the subject's clinical and laboratory variables according to the published and available PGA from ImproveCareNow accessible on their website and Table 1: abdominal pain, diarrhea, bloody stools, fatigue, activity level, weight loss, abdominal masses or tenderness, toxic appearance, presence of perianal fistula, development or worsening anemia, elevated ESR/CRP, and hypoalbuminemia.^15^ Patients were considered to have a complication at a time point if they were HACA‐positive, had an infusion reaction, were nonresponsive to IFX, developed cancer, or developed a psoriaform rash. The reason for stopping IFX, if applicable, was recorded for all patients.

**Table 1:** ImproveCareNow physician global assessments†, 15

**Inactive disease**	In the past week the patient has had minimal or no symptoms thought to be secondary to IBD
Abdominal pain Diarrhea, bloody stools Fatigue, Activity	Asymptomatic Mild symptoms on one or two occasions that resolved spontaneously Significant symptoms felt to be secondary to another disorder such as IBS or depression
Fistula	None, or a noninflamed, indolent fistula with no or minimal drainage
Weight Loss	No unexplained weight loss
Abd mass, tenderness	None
Toxic appearance	No
Lab tests (if available)	Normal or minimal transient abnormalities
**Mild disease**	In the past week, the patient has had mild recurring or persistent symptoms thought to be secondary to IBD
Abdominal pain	Mild abdominal pain thought to be secondary to IBD and occurring several times a week
Diarrhea, bloody stools	Mild recurrent diarrhea (without nocturnal defecation or gross inflammatory bleeding) thought to be secondary to IBD
Fatigue Activity	Asymptomatic or mild symptoms on one or two occasions that resolved spontaneously Significant symptoms felt to be secondary to another disorder such as IBS or depression
Fistula	Active fistula or other perianal symptoms without associated symptoms
Weight loss	No unexplained weight loss
Abd mass, tenderness	None
Toxic appearance	No
Lab tests (if available)	Persistent (and significant) laboratory abnormalities felt to be secondary to IBD with no or mild associated symptoms
**Moderate disease**	In the past week, the patient has had moderate (or combinations of mild and moderate) recurring or persistent symptoms thought to be due to IBD
Abdominal pain	Moderate abdominal pain thought to be secondary to IBD
Diarrhea, bloody stools	Moderate diarrhea that could include nocturnal diarrhea and gross inflammatory bleeding thought to be secondary to IBD
Fatigue	Significant fatigue thought to be secondary to IBD
Activity	Inability to maintain normal activities due to fatigue or other symptoms
Fistula	Active fistula or other perianal disease in combination with other symptoms
Weight loss	Significant unexplained weight loss
Abd mass, tenderness	Abdominal tenderness and/or small abdominal mass or fullness
Toxic appearance	No
Lab tests (if available)	Significant anemia, hypoalbuminemia, and/or elevation in inflammatory markers
**Severe disease**	In the past week, the patient has had severe (or combinations of moderate and severe) recurring or persistent symptoms thought to be due to IBD
Abdominal pain	Severe abdominal pain thought to be secondary to IBD
Diarrhea, Bloody stools	Significant diarrhea that could include nocturnal diarrhea and gross inflammatory bleeding thought to be secondary to IBD
Fatigue	Significant fatigue thought to be secondary to IBD
Activity	Severe impairment of normal activities due to fatigue or other symptoms
Fistula	Active fistula or other perianal disease in combination with other symptoms
Weight loss	Significant unexplained weight loss
Abd mass, tenderness	Abdominal mass and/or tenderness
Toxic appearance	Appears toxic
Lab tests (if available)	Significant anemia, hypoalbuminemia, and/or elevation in inflammatory markers

† © Nationwide Children's Hospital, 2017. Modified and reproduced with permission.

IBD, inflammatory bowel disease.

### *Statistical analysis*

All analyses were calculated using SAS v9.4. Univariate and bivariate analysis was conducted to describe the characteristics of and a subset of outcomes experienced by the patient sample overall and by IBD type (CD or UC). Patients with indeterminate IBD types were excluded from all analysis. Chi‐square tests for categorical variables and independent samples *t*‐tests with a Satterthwaite approximation were used for continuous variables to determine if any characteristics differed by IBD type. For strongly skewed variables, a Wilcoxon rank‐sum test was used.

Separate logistic regression models were constructed to examine which patient characteristics differed by PE. These models were repeated for all three PE outcomes—PE at specified visits, PE at specified visits plus visits around adverse outcomes, and PE at any visit. Univariate analysis was used to describe the proportion of patients that experienced complications of IFX therapy and the proportion of patients that stopped taking IFX.

We examined whether patients on IFX with PE at any time are more likely to have adverse events and whether patients with more frequent PE (≥2, 1, never) are more likely to have adverse events. A generalized linear mixed model with a binary distribution, penalized quasi‐likelihood estimates, and random terms for each subject was used to assess this relationship. The adjusted models included the following potential confounders: race, sex, age at diagnosis, IBD type, and ever steroid use during the study.

Lastly, repeated measures logistic regression with an exchangeable correlation structure adjusted for race (African American, Caucasian, other), sex, age at diagnosis (continuous), IBD type, and steroid use (yes/no treated as time‐varying) was used to determine if, among patients using IFX, mild or greater disease severity is more likely to co‐occur with visits where patients have PE. The adjusted model was run restricting to the specified visits and subsequently for the specified plus adverse outcome visits. There was no disease severity data available at visits other than specified and adverse outcome visits.

## Results

In total, 122 patients with IBD starting IFX were identified for this study. One patient with an indeterminate IBD type was excluded from all analysis for a final analytic sample of 121 patients. The mean age at diagnosis was 12.4 years (SD = 3.48, range = 4–22 years, 3 patients 18–22 years), 55.3% of patients were male (n = 67), and 69.4% (n = 84) were Caucasian. Further descriptive study population data stratified by IBD type are listed in Table 2.

**Table 2:** Descriptive characteristics of pediatric patients stratified by IBD type (*N* = 121)

	IBD type		
Characteristic	CD (*n* = 108)	UC (*n* = 13)	*P*‐value
Age at diagnosis in years, mean (SD)	12.4 (3.40)	12.3 (4.27)	0.95‡
Male, *n* (col %)	59 (54.6)	8 (61.5)	0.64§
Race, *n* (col %)			0.01¶
Caucasian	76 (70.4)	8 (61.5)	
African American	27 (25.0)	2 (15.4)	
Other	5 (4.6)	3 (23.1)	
Phenotype			0.12¶
Inflammatory	70 (64.8)	13 (100.0)	
Stricturing	13 (12.0)	0 (0.0)	
Penetrating	16 (14.8)	0 (0.0)	
Stricturing/penetrating	9 (8.3)	0 (0.0)	
Disease location—esophagus, *n* (col %)	25 (23.2)	0 (0.0)	0.07¶
Disease location—stomach, *n* (col %)	68 (63.0)	0 (0.0)	<0.01§
Disease location—duodenum, *n* (col %)	66 (61.1)	0 (0.0)	<0.01§
Disease location—TI, *n* (col %)	83 (76.9)	0 (0.0)	<0.01¶
Disease location—colon, *n* (col %)	96 (88.9)	13 (100.0)	0.36¶
Disease location—small bowel, *n* (col %)	2 (1.9)	0 (0.0)	1.00¶
Disease location—perianal, *n* (col %)	12 (11.1)	0 (0.0)	0.35¶
Atopic conditions,† *n* (col %)	41 (38.0)	2 (15.4)	0.13¶
Years since diagnosis at IFX start, median (IQR)	0.5 (1.75)	0.3 (1.08)	0.24††
Surgery prior to IFX, *n* (col %)	12 (11.1)	0 (0.0)	0.36¶
Time on IFX in study in years, mean (SD)	3.0 (2.13)	4.1 (2.55)	0.15‡
Dose escalation required, *n* (col %)	40 (37.0)	6 (46.2)	0.56¶
Time to dose escalation in years, mean (SD)	1.1 (1.02)	2.0 (2.96)	0.51‡
Number of flares on IFX, mean (SD)	0.8 (1.17)	1.8 (1.91)	0.07‡
Hospitalization required on IFX, *n* (col %)	24 (22.2)	7 (53.9)	0.02¶
PE at specified visit, *n* (col %)	21 (19.4)	6 (46.2)	0.07¶
PE at specified + adverse outcome visit, *n* (col %)	26 (24.1)	6 (46.2)	0.10¶
PE at “any visit”, *n* (col %)	36 (33.3)	8 (61.5)	0.08¶
Frequency of PE, mean (SD)	0.7 (1.24)	2.2 (2.04)	0.02‡

† Atopic patients were identified as those with asthma, eczema, psoriasis, seasonal allergies, or food allergies.

‡ *t*‐test—Satterthwaite.

§ Chi‐square.

¶ Fishers.

†† Wilcoxon.

IBD, inflammatory bowel disease; IFX, Infliximab; PE, peripheral eosinophilia.

Patients spent an average of 3.1 years (SD = 2.20, 1 month to 10.2 years) on IFX during the study period and 38.0% required a dose increase on average 1.3 years (SD = 1.40) after initiation of IFX. Patients experienced an average of 0.9 disease exacerbations (SD = 1.30, range = 0–7) and 31 (25.6%) required a hospitalization while receiving IFX. Twenty‐seven (22.3%) patients had PE at specified study visits, 32 (26.5%) had PE when including adverse outcome visits, and 44 (36.4%) had a PE at any study visit. On average, patients experienced 0.8 (SD = 1.41, range = 0–6) unique episodes of PE. The relationship between the various patient characteristics and PE is presented in Table 3. Younger age at diagnosis of pediatric IBD was an independent risk factor for development of PE while having UC was an independent risk factor only at the time of “adverse outcome” visits. Female gender was negatively associated with the incidence of PE, but only when analyzed for all visits.

**Table 3:** Independent association between patient characteristics and peripheral eosinophilia (PE) at specified visits, PE at specified + adverse outcome visits, or PE at “any visit” (*N* = 121)

	PE at specified visits		PE at specified + adverse outcome visits		PE at “any visit”	
Independent predictor	OR	95% CI	OR	95% CI	OR	95% CI
IBD type						
Crohn's disease	0.28	(0.09, 0.93)	0.37	(0.11, 1.20)	0.34	(0.10, 1.11)
Ulcerative colitis	1.0	REF	1.0	REF	1.0	REF
Age at diagnosis in years	0.86	(0.76, 0.97)	0.86	(0.75, 0.97)	0.86	(0.76, 0.97)
Sex						
Male	1.84	(0.75, 4.50)	2.15	(0.92, 5.06)	2.61	(1.20, 5.67)
Female	1.0	REF	1.0	REF	1.0	REF
Race						
Caucasian	0.82	(0.15, 4.40)	1.07	(0.20, 5.67)	1.08	(0.24, 4.82)
African American	0.96	(0.16, 5.85)	1.14	(0.19, 6.88)	0.88	(0.17, 4.45)
Other	1.0	REF	1.0	REF	1.0	REF
Phenotype						
Inflammatory	2.71	(0.32, 22.96)	3.45	(0.41, 29.04)	1.46	(0.34, 6.23)
Stricturing	0.67	(0.04, 12.27)	1.45	(0.11, 18.95)	0.36	(0.05, 2.82)
Penetrating	2.67	(0.25, 28.44)	2.67	(0.25, 28.43)	1.20	(0.22, 6.68)
Stricturing/penetrating	1.0	REF	1.0	REF	1.0	REF
Disease location—esophagus	1.13	(0.40, 3.18)	1.41	(0.54, 3.68)	1.37	(0.56, 3.34)
Disease location—stomach	1.42	(0.62, 3.25)	1.42	(0.62, 3.26)	1.18	(0.56, 2.48)
Disease location—duodenum	1.87	(0.81, 4.32)	1.87	(0.81, 4.32)	1.75	(0.83, 3.71)
Disease location—TI	0.57	(0.25, 1.33)	0.57	(0.25, 1.33)	0.66	(0.30, 1.45)
Disease location—colon	NE	XX	NE	XX	3.38	(0.71, 16.20)
Disease location—small bowel	NE	XX	NE	XX	1.64	(0.10, 26.95)
Disease location—perianal	2.83	(0.82, 9.75)	2.17	(0.64, 7.40)	1.73	(0.52, 5.71)
Atopic conditions†	0.89	(0.39, 2.06)	0.89	(0.39, 2.06)	0.89	(0.39, 2.06)
Years since diagnosis at IFX start	0.94	(0.77, 1.15)	0.94	(0.77, 1.15)	0.94	(0.77, 1.15)
Surgery prior to IFX	1.90	(0.39, 9.18)	1.90	(0.39, 9.18)	1.90	(0.39, 9.18)

† Atopic patients were identified as those with asthma, eczema, psoriasis, seasonal allergies, or food allergies.

IBD, inflammatory bowel disease; IFX, Infliximab.

After adjusting for confounding variables, patients with PE had higher odds of having clinically active disease when examining specified (OR = 2.65, 95% CI: 1.31, 5.34) as well as specified plus adverse event visits (OR = 2.31, 95% CI: 1.40, 3.81).

In total, 29 patients (24.0%) experienced at least one complication while taking IFX; some of these patients may have had additional complications. Fourteen (11.6%) were HACA‐positive, 5 (4.1%) experienced an infusion reaction, 10 (8.3%) had a loss of response to IFX, 1 (0.8%) developed cancer, and 3 (2.5%) developed a psoriaform rash. Only two patients stopped taking infliximab due to patient preference.

There was no association between having PE and any individual complication (OR = 1.00, 95% CI: 0.42, 2.35) (Table 4). Regarding overall frequency of PE events, 19 patients (15.7%) had one unique PE event and 25 (20.7%) patients had two or more unique PE events. There was no association between the number of PE events and adverse outcomes. Patients with ≥2 unique PE events had 1.04 times the odds of an adverse outcome (95% CI: 0.36, 2.98) and patients with 1 unique PE event had 1.17 times the odds of a complication (95% CI: 0.37, 3.70), compared to patients with no PE events. There was no association between the frequency of PE with any specific adverse outcome (HACA+, infusion reaction, nonresponse, cancer, or psoriatic rash). The same findings were seen in the models adjusted for race, sex, age at diagnosis, IBD type, and any steroid use during the study (not included in tables).

**Table 4:** Association between peripheral eosinophilia (PE) and complications

	Adverse Event (All)		HACA+		Infusion Reaction		Nonresponse		Cancer		Psoriatic Rash	
	OR	95% CI	OR	95% CI	OR	95% CI	OR	95% CI	OR	95% CI	OR	95% CI
PE “any visit”	1.00	(0.42, 2.35)	1.26	(0.41, 3.88)	1.09	(0.18, 6.79)	0.68	(0.17, 2.76)	NE	XX	0.81	(0.07, 9.20)
Never had PE	1.00	REF	1.00	REF	1.00	REF	1.00	REF	1.00	REF	1.00	REF
≥2 PE	1.04	(0.36, 2.98)	1.64	(0.45, 6.00)	1.03	(0.10, 10.35)	0.42	(0.05, 3.56)	NE	XX	NE	XX
1 PE	1.17	(0.37, 3.70)	1.02	(0.20, 5.22)	1.37	(0.14, 13.96)	1.18	(0.22, 6.18)	NE	XX	2.08	(0.18, 24.26)
Never had PE	1.00	REF	1.00	REF	1.00	REF	1.00	REF	1.00	REF	1.00	REF

NE, not estimable (quasi‐separation of data points).

## Discussion

The gastrointestinal tract, due to its interactions with its microenvironment, has eosinophils present to some degree in the lamina propria. Primary eosinophilic disorders of the GI tract such as eosinophilic esophagitis, food protein‐induced enterocolitis syndrome, eosinophilic gastroenteritis, and other eosinophilic gastroenteritides are characterized by eosinophilic infiltration and inflammation in the absence of other causes of eosinophilia such as parasites, drug reactions, and neoplasms.^3^, ^4^, ^5^ IBD is characterized by intestinal infiltration of inflammatory cells of which eosinophils have also been noted.^6^ As IL‐5 stimulates eosinophil release into peripheral circulation and eotaxin draws activated eosinophils into the GI mucosa along with TNF‐alpha, eosinophils become part of the inflammatory milieu.^16^, ^17^, ^18^ Eosinophilic degranulation has been associated with fibroblast proliferation in CD which may be a contributor to stricture formation.^19^

Based on our study results, the development of PE was an independent risk factor for clinically active disease. Unlike the study by Sadi *et al*. our results did not correlate with the degree of clinical severity based on the PGA clinical scale we used. This association between PE events and increased disease activity suggests that eosinophilia may serve as an additional biomarker of disease control as reported by other investigators.^12^, ^20^ Our study also found no correlation between PE and disease severity immediately prior to starting IFX or during treatment although our patient sample size may impact this outcome. The design of this study with no separate control cohort of pediatric IBD patients not treated with IFX precludes any additional comparisons or conclusions as this was not the primary aim of the study. Furthermore, the presence of or the degree of PE was not compared with accepted inflammatory markers such as the erythrocyte sedimentation rate, C‐reactive protein, or stool calprotectin.

Regarding our primary endpoint, the analysis of our data demonstrates that the occurrence of PE in IBD patients on IFX at our center was not associated with adverse outcomes, including the loss of IFX efficacy or the development of HACA. PE in these patients was also not associated with adverse outcomes of psoriasis, cancer, or the development of infusion reactions. However, the limited occurrence of these complications in our patients may also limit the strength of this conclusion. The overall frequency of PE events in these patients also did not affect adverse outcomes.

A limitation of our study includes our definition of PE as this varies from the World Health Organization classification, which requires multiple values >500 cells/μL 4–8 weeks apart. This discrepancy was made to assess response to isolated eosinophilia measurements as an exposure, which would not be captured by the WHO definition and to better assess the effect of PE frequency rather than looking for persistent PE as per the WHO definition. Both the WHO definition and study definition recognize that the percentage of eosinophils in peripheral blood is relative as this can be affected by numerous factors, including leukopenia or relative changes in the ratio of other leukocyte subtypes.

During data analysis of adverse events by IBD patients on IFX with and without PE was performed by a simple power calculation based on chi‐squared test. It was found that given our sample size and the rate of adverse events in patients without PE (24%), we had 74% power to detect a 20% increase in adverse events and 88% power to detect a 25% increase in adverse events in patients with PE. As a single‐center study, the power to detect these differences in our population is a limitation. For further detection of differences, a multicenter study or IBD research consortium would be necessary.

Other limitations of this study include that our IBD population on IFX was primarily patients with CD (88.5%). However, it is unclear whether this is an abnormal proportion as this varies greatly by region, by pediatric age groups, and as the incidence of CD is rising faster than that of UC.^21^, ^22^, ^23^, ^24^ Our statistical analysis adjusted for IBD type, and our results were consistent despite the predominance of CD patients. This was also a retrospective study and we had limitations relating to data, as documentation was noted to be inconsistent across this chart review such that a research validated disease activity index (Pediatric Crohn's Disease Activity Index (PCDAI) and the Pediatric Ulcerative Colitis Activity Index (PUCAI) used in most pediatric IBD studies) could not be calculated and consequently a PGA was required instead. A prospective study, and one that allows for complete data collection and use of a more continuously scaled global assessment scale such as the PCDAI and PUCAI may also better document any relationship between eosinophilia and disease control.

Currently there is no universal standard of care for routine therapeutic drug monitoring of IFX‐ and patient‐centered dosing, although such may prove useful and the initiative is being discussed at international IBD conferences. While parasitic infections can also cause PE, this study could not exclude this possibility as ova and parasites stool studies are not routinely ordered even in immunocompromised subjects since the incidence of parasitic infection is low in the developed world. In a prospective study, this factor could be controlled for with stool ova and parasite collection. Methotrexate and mesalamines also have been linked with tissue eosinophilia, but are not routinely linked with PE in the IBD literature and are unlikely to have significantly biased our results. Finally, it is possible that an association between eosinophilia and adverse events in pediatric IBD patients on IFX exists but that we were unable to detect this due to our study sample size.

In conclusion, PE in IBD patients on IFX treated at this pediatric IBD Center was found to be a risk factor for clinically active disease but was not found to be associated with adverse outcomes or loss of IFX efficacy. Furthermore, we found that time on IFX at standard (5 mg/kg every 8 weeks) or high dose (10 mg/kg or 5 mg/kg sooner than every 8 weeks) was not associated with increases in mean AEC, suggesting that IFX is not associated with PE. Further studies with a larger sample size to account for smaller differences would be needed to confirm this finding and better determine if there is an association between AEC and disease severity. However, at this time, our study suggests that PE is not an adequate marker to help predict adverse outcomes of IFX use in pediatric IBD.
